# Prospective assessment of the predictive value of the *BRCA1* gene status in sarcoma patients treated with trabectedin: an updated analysis of the EORTC 62091 trial

**DOI:** 10.1002/cam4.1403

**Published:** 2018-04-15

**Authors:** Antoine Italiano, Nathan Touati, Saskia Litière, Françoise Collin, Philippe Pourquier, Alessandro Gronchi

**Affiliations:** ^1^ Institut Bergonié Bordeaux France; ^2^ INSERM U1218 Bordeaux France; ^3^ European Organization for Research and Treatment of Cancer Brussels Belgium; ^4^ Centre Georges‐François Leclerc Dijon France; ^5^ Institute of Cancer Research of Montpellier Montpellier France; ^6^ Fondazione IRCCS Istituto Nazionale dei Tumori Milano Italy

## Abstract

We describe the predictive value of BRCA1 gene status on trabectedin efficacy and found no correlation despite the mechanisms of action of this drug that rely on DNA repair systems.

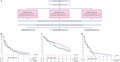

Trabectedin is approved for the treatment of patients with advanced soft tissue sarcoma (STS) after the failure of anthracyclines and ifosfamide, or when they are not eligible to receive these agents. In this setting, the 6‐month progression‐free rate is about 35–40% 1. The identification of predictive biomarkers of the clinical benefit of trabectedin in STS patients is thus a crucial issue to identify potential responders.

Previous in vitro studies have demonstrated that trabectedin cytotoxicity depends on the status of both nucleotide excision repair (NER) and homologous recombination (HR) DNA repair pathways and that cells deficient in HR were more sensitive to trabectedin than their nondeficient counterparts 2. *BRCA1*, a key regulator involved in DNA‐ends resection during HR, was part of the gene expression signature associated with sensitivity to trabectedin in human sarcoma cells explanted from patients not previously treated by chemotherapy 3. Several retrospective clinical pharmacogenomic studies suggested that BRCA1 status may be predictive of trabectedin efficacy in sarcoma patients 4, 5.

We report here the first study assessing the predictive value of this biomarker in a context of a randomized controlled trial. The aim of the EORTC 62091 trial was to compare the efficacy of trabectedin to doxorubicin in the first‐line setting of advanced/metastatic soft tissue sarcoma. Although the trial was stopped early due to lack of efficacy of trabectedin as compared to doxorubicin, several patients experienced a long duration of benefit to trabectedin. Endpoints have been updated with more complete follow‐up for the purpose of this study.

In this randomized multicentre prospective dose‐selection phase IIb superiority trial, 133 patients were randomized between doxorubicin (*n* = 43), trabectedin (3‐h infusion, *n* = 47), and trabectedin (24‐h infusion, *n* = 43). Based on tumor samples availability, BRCA1 genotype status was available for 60 patients (Fig. 1A). Haplotype was defined as previously described 5. Patients with BRCA1 haplotype being at least one AAAG allele have been classified into the “favorable” group and the other into the “nonfavorable” group.

**Figure 1:**
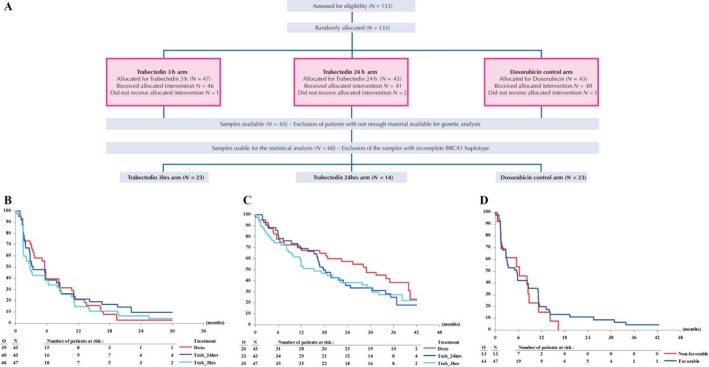
(A) Flowchart of the TRUSTS trial/BRCA1 haplotype research project. (B) Duration of progression‐free survival by treatment. (C) Duration of overall survival by treatment. (D) Duration of progression‐free survival by BRCA1 haplotype.

At the time of this analysis, one patient was still on protocol treatment. Major reasons for protocol treatment discontinuation were disease progression (12/40 doxo, 26/40 trab24 h, 31/46 trab3 h) and toxicity (1/40 doxo, 8/40 trab24 h, 7/46 trab3 h). No significant improvement was observed in the trabectedin arms as compared to the doxorubicin arm in PFS (Table 1, Fig. 1B) and OS (Table 1, Fig. 1C).

**Table 1:** Progression‐free survival/Overall survival according to treatment arm and BRCA1 haplotype (Cox regressions)

	Patients (N)	Observed events (O)	Hazard ratio (95% CI)	One‐sided *P*‐value (Log‐rank)	Median (95% CI) (Months)
Progression‐free survival—treatment					
Doxorubicin	43	39	1.00		5.52 (3.12, 8.28)
Trabectedin 24 h	43	40	**0.90** (0.57, 1.40)	0.317	3.35 (2.60, 8.48)
Trabectedin 3 h	47	46	**1.11** (0.72, 1.71)	0.687	2.76 (1.45, 6.18)
Overall survival—treatment					
Doxorubicin	43	26	**1.00**		28.91 (16.82, 39.92)
Trabectedin 24 h	43	33	**1.38** (0.83, 2.32)	0.892	18.23 (15.28, 24.77)
Trabectedin 3 h	47	35	**1.44** (0.87, 2.40)	0.921	15.31 (9.43, 27.83)

				Two‐sided *P*‐value (Log‐rank)	Interaction test *P*‐value
Progression‐Free survival—sBRCA1 haplotype					
Nonfavorable	13	13	**1.00**	0.509	0.902
Favorable	47	44	**0.81 (0.43, 1.52)**		

There was no statistically significant association between BRCA1 haplotype and PFS (Table 1, Fig. 1D).

Trabectedin was not shown to be superior to doxorubicin in this setting and therefore, doxorubicin remains the standard first‐line treatment of advanced STS. The predictive value of BRCA1 haplotype for trabectedin efficacy suggested by retrospective studies could not be confirmed in this cohort. This may be related to the limited number of patients with available tumor samples but could also be due to the absence of *BRCA1* expression levels assessment. Indeed, RNA extracted from formalin‐fixed paraffin‐embedded (FFPE) tissue is problematic due to chemical modifications and continued degradation overtime. We believe that the identification of a genomic DNA‐based signature would be more adapted to routine practice than biomarkers including RNA expression data. RNA is less stable than DNA and consensual definition of cutoff levels for low or high expression is challenging. Moreover, trabectedin not only has direct effects against cancer cells but also has host‐modulating properties that appear to be of great importance for its therapeutic effect 6. Strong preclinical and clinical evidence reveals the ability of this drug to decrease the number of tumor‐associated macrophages and to modify the tumor microenvironment and angiogenesis at therapeutically relevant doses. Therefore, it seems plausible to hypothesize that the multiple mechanisms of action may have different roles in different tumors, and thus that a unique biomarker will not be able to select accurately the patients who are more likely to benefit from this drug.

## Conflict of Interest

The authors declare that they have no competing interests.

## Ethics Approval and Consent to Participate

This study was approved by the ethics committee of the Comprehensive Cancer Center Institut Bergonié (Bordeaux, France).

## Availability of Data and Material

The datasets supporting the conclusions of this article cannot be shared for confidentiality reasons.
